# Perceptions and behaviors related to hand hygiene for the prevention of H1N1 influenza transmission among Korean university students during the peak pandemic period

**DOI:** 10.1186/1471-2334-10-222

**Published:** 2010-07-28

**Authors:** Jae-Hyun Park, Hae-Kwan Cheong, Dae-Yong Son, Seon-Ung Kim, Chang-Min Ha

**Affiliations:** 1 Department of Social and Preventive Medicine, Sungkyunkwan University College of Medicine, Suwon, Korea; 2 Sungkyunkwan University College of Medicine, Suwon, Korea

## Abstract

**Background:**

This study was performed to better assess the perceptions, motivating factors, and behaviors associated with the use of hand washing to prevent H1N1 influenza transmission during the peak pandemic period in Korea.

**Methods:**

A cross-sectional survey questionnaire was completed by 942 students at a university campus in Suwon, Korea, between December 1 and 8, 2009. The survey included questions regarding individual perceptions, motivating factors, and behaviors associated with hand washing for the prevention of H1N1 influenza transmission.

**Results:**

Compared to one year prior, 30.3% of participants reported increasing their hand washing frequency. Female students were more likely to practice more frequent hand washing. Women also perceived the effectiveness of hand washing to be lower, and illness severity and personal susceptibility to H1N1 infection to be higher. Study participants who were female (OR: 1.79-3.90) who perceived of hand washing to be effective (OR: 1.34-12.15) and illness severity to be greater (OR: 1.00-3.12) washed their hands more frequently.

**Conclusions:**

Korean students increased their frequency of hand hygiene practices during the pandemic, with significant gender differences existing in the attitudes and behaviors related to the use of hand hygiene as a means of disease prevention. Here, the factors that affected hand washing behavior were similar to those identified at the beginning of the H1N1 or SARS pandemics, suggesting that public education campaigns regarding hand hygiene are effective in altering individual hand hygiene habits during the peak periods of influenza transmission.

## Background

In April 2009, a new strain of influenza virus - A/H1N1 - began appearing in several different countries around the world. The World Health Organization (WHO) raised the influenza pandemic alert level to Phase 5 on April 29, 2009, and later to Phase 6, indicating that a full global pandemic was under way. By May 23, 2009, 12,022 H1N1 cases and 83 deaths had been confirmed in 43 countries [1]. In Korea, the first H1N1 influenza infection case was confirmed on May 3, 2009, and the first H1N1-related death occurred on August 15, 2009. By the beginning of September 2009, H1N1 cases had increased dramatically, with 4,293 cases and 3 deaths confirmed by the end of September 2009. The H1N1 infection rate peaked two months later in November 2009, at which time approximately 9,000 new cases of A/H1N1 influenza were being confirmed each day. By the end of the month, a total of 104 deaths had resulted from H1N1-related causes [2], leading the Korean government to declare a public "Emergency Response Level." At that time, approximately one half of all patients seeking treatment for "common cold-like symptoms" were found to have H1N1 influenza[3].

As no vaccine for H1N1 Influenza had been developed, the Korean government attempted to mitigate the spread of disease by reducing transmission. Infected individuals were identified, isolated, and treated, while public health campaigns were initiated to educate the general public about preventive behaviors that reduce the risk of transmission. The specific techniques recommended included sneezing into a tissue and washing hands regularly with soap and water [4]. Given that the evidence regarding the preventive effectiveness of wearing a facemask is inconclusive [5], hand hygiene was considered the easiest and most effective measure [6] to prevent the spread of influenza A/H1N1.

Previously, multiple studies were undertaken to identify the factors that most effectively motivated people to adopt specific hand hygiene behaviors. Data collected during the SARS pandemic and at the onset of the H1N1 epidemic had already ascertained several such factors, including the perceived effectiveness of hand hygiene [7,8], the perceived susceptibility to illness [8-10], the perceived severity of the illness [8], and levels of anxiety associated with infection [7,11]. However, most of the previous studies were preformed during the recent SARS pandemic, and several studies were conducted at the outset of the H1N1 influenza pandemic [12,13].

The current study was designed to qualify recent hand-washing behaviors, changes in hand-washing behaviors, relationship between hand washing frequency and flu-like symptom as well assess the perceptions, attitudes, and motivating factors regarding hand washing during the peak period of the H1N1 influenza pandemic through the use of a cross-sectional survey model.

## Methods

### Participants and Procedures

For this study, subjects were recruited by convenience sampling from Sungkyunkwan University, a public university located in Suwon, Korea with an enrollment of 8,485 male students and 2,600 female students. Inclusion criteria included current enrollment as a student and willingness to participate in a research study. Without exception, all students who agreed to participate completed the questionnaire. Subjects were recruited from university residence halls and the campus library during a one-week period between 12/01/09 and 12/8/09. Individuals who agreed to participate were given the study questionnaire to complete on their own. In total, 942 students (738 males, 204 females) completed the questionnaire, with the proportion of male participants to female participants accurately reflecting the student body demographics at Sungkyunkwan University. Pilot surveys were conducted prior to the study in order to confirm that participants could understand the survey questions and to ensure the validity of the questionnaire content. with face or content validity of the questionnaires. Using the results of this pilot study, the survey questionnaire was amended to create a final version. This study was approved by the Samsung Medical Center IRB (No: 2009-12-030) in Korea.

### Measurements

The study questionnaire was designed to assess recent hand-washing behaviors, changes in hand-washing behaviors, information encountered regarding hand washing, perceived effectiveness of hand washing in preventing infection with H1N1 influenza, perceived severity of H1N1 influenza, perceived susceptibility to H1N1 influenza infection, and recent flu-like symptoms. The study questionnaire was modeled after other similar questionnaires used in prior SARS or H1N1 studies [12,13], and was subsequently modified to reflect the current situation of the H1N1 epidemic in Korea. The variables, matched questions, and answer categories included in the survey questionnaire (see Additional file 1) are summarized in Table 1. Subject gender, age, and residence type (university residence hall versus other) was also recorded. Additionally, participants were asked if any acquaintances (e.g. family members, friends, etc) were currently experiencing flu-like symptoms.

**Table 1:** Variables and matched questions in the questionnaire

Variables	Questions	Answer category
Daily hand washing frequency during the past month	How many times per day did you wash your hands with soap during the previous month?	#1, 2-4, 5-7, 8-9, #10

Daily hand washing frequency one year ago	How many times per day did you wash your hands with soap one year ago?	#1, 2-4, 5-7, 8-9, #10

Information encountered	Have you seen or heard any information regarding hand washing as a prevention strategy for H1N1 influenza transmission?	Yes, No

Perceived effectiveness	Do you consider hand washing to be an effective means of preventing H1N1 influenza infection?	Negligible, More or less, Substantial

Perceived severity	If you were infected with H1N1 influenza, how great of a burden would that be on your daily life?	Mild symptom like common cold, Substantial limitation in daily life, Have severe consequences, May die from it

Perceived susceptibility	How possible do you believe it is for you to become infected with H1N1 influenza?	Very low, Somewhat low, Nor low, nor high, Somewhat high, Very high

Recent flu symptoms in participant	Have you recently experienced any flu-like symptoms?	Yes, No

Recent flu symptoms in acquaintances	Do you have any acquaintances who experienced any flu-like symptoms recently?	Yes, No

### Statistical analyses

Using chi-squared tests, the following variables were examined for differences by gender: recent hand washing frequency, changes in hand washing frequency, information encountered about hand washing, perceived effectiveness of hand washing, perceived severity of H1N1 influenza, and perceived susceptibility to H1N1 infection, and recent flu-like symptoms. Additionally, each variable was evaluated for correlation with all other variables using the chi-squared test to identify trends. To identify factors related to the frequency of hand washing, both chi-square tests and multiple logistic regression models were used. For the logistic regression modeling, additional independent variables were selected based on data from several previous studies, including gender, presence flu-like symptoms among subject acquaintances, information encountered about hand washing, perceived effectiveness of hand washing, perceived illness severity, and perceived illness susceptibility. Chi-squared tests were also used to evaluate the relationship between recent hand washing frequency and recent flu-like symptoms by gender, with multiple logistic regression modelling employed to adjust for gender, age, type of residence, and presence of flu-like symptoms among acquaintances. Recent hand washing frequency was re-classified #4, 5-7, #8, given the frequency of each category and statistical stability in multiple logistic regression models. All of the multiple logistic regression models were validated by the average receiver operating characteristics and the *p*-value for the Hosmer-Lemeshow goodness-of-fit test. The software program SPSS 15.0 was used to for all data analysis. Two-tailed *p*-values ≥ 0.05 were considered statistically significant.

## Results

### General study subject demographics

General participant demographics are outlined in Table 2. A total of 942 participants were enrolled, of which 78.3% were male. Male students were more likely to live in university residence halls, while more female students were more likely to have acquaintances that had experienced flu-like symptoms in the recent past.

**Table 2:** General demographics, reported hand washing behaviors, and perceived effectiveness, severity, and susceptibility of the participants

	Total		Males		Females		P-value*
				
	N	(%)	N	(%)	N	(%)	
Total	942	(100.0)	738	(78.3)	204	(21.7)	-
Age group							
18-19	244	(26.3)	178	(24.5)	66	(32.8)	<0.001
20-21	214	(23.1)	157	(21.6)	57	(28.4)	
22-23	236	(25.4)	182	(25.0)	54	(26.9)	
24+	234	(25.2)	210	(28.9)	24	(11.9)	
Type of residence							
University residence halls	530	(56.4)	435	(59.2)	95	(46.6)	0.001
Others	409	(43.6)	300	(40.8)	109	(53.4)	
Acquaintances with recent "flu-like" symptoms							
No	380	(40.3)	324	(43.9)	56	(27.5)	<0.001
Yes	562	(59.7)	414	(56.1)	148	(72.5)	
Recent "flu-like" symptoms							
No	685	(72.7)	568	(77.0)	117	(57.4)	<0.001
Yes	257	(27.3)	170	(23.0)	87	(42.6)	
Daily hand washing frequency							
#1	68	(7.2)	66	(8.9)	2	(1.0)	<0.001
2-4	503	(53.4)	418	(56.6)	85	(41.7)	
5-7	240	(25.5)	165	(22.4)	75	(36.8)	
8-9	70	(7.4)	49	(6.6)	21	(10.3)	
#10	61	(6.5)	40	(5.4)	21	(10.3)	
Information encountered							
No	76	(8.1)	65	(8.8)	11	(5.4)	0.113
Yes	866	(91.9)	673	(91.2)	193	(94.6)	
Perceived effectiveness							
Negligible	40	(4.3)	32	(4.3)	8	(3.9)	0.006
More or less	300	(31.9)	216	(29.3)	84	(41.2)	
Substantial	600	(63.8)	488	(66.3)	112	(54.9)	
Perceived severity							
Mild symptoms like common cold	526	(56.1)	439	(59.7)	87	(42.9)	<0.001
Substantial limitation in daily life	297	(31.7)	214	(29.1)	83	(40.9)	
Have severe consequences	29	(3.1)	25	(3.4)	4	(2.0)	
May die from it	86	(9.2)	57	(7.8)	29	(14.3)	
Perceived susceptibility							
Very low	192	(25.9)	169	(28.7)	23	(14.9)	0.002
Somewhat low	249	(33.6)	200	(34.0)	49	(31.8)	
Nor low, nor high	244	(32.9)	178	(30.3)	66	(42.9)	
Somewhat high	44	(5.9)	31	(5.3)	13	(8.4)	
Very high	13	(1.8)	10	(1.7)	3	(1.9)	

* chi-square test

### Changes in hand washing frequency

**Figure 1:**
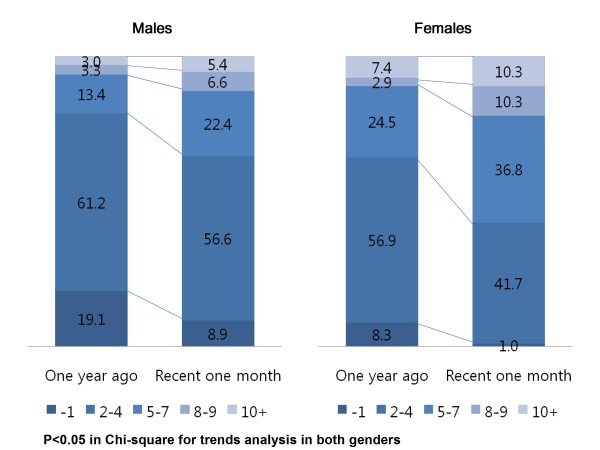
**Changes in reported daily hand washing frequency (%)**.

The reported frequency of hand washing behavior was greater in women than men (Table 2). While 34.4% of male students reported washing their hands five times a day, 57.4% of female students reported washing their hands with this same frequency. Additionally, 30.3% of participants reported increasing their personal frequency of hand washing within the last year. After stratifying by gender, both groups reported an increased frequency of hand washing compared with one year ago, with these trends reaching statistical significance by Chi-square analysis (Figure 1).

### Information encountered and perceptions related to the effectiveness of hand washing, severity of the illness, and susceptibility to H1N1 influenza

A majority of participants reported encountering information regarding hand washing (males: 91.2%, females: 94.6%) and perceived hand washing as an effective measure to prevent H1N1 influenza transmission (Table 2). Men were more likely to perceive hand washing as an effective preventive measure (P = 0.006). Regarding perception of H1N1 influenza severity, while 56.1% of subjects believed that infection with H1N1 influenza would produce mild symptoms similar to those of the common cold, women were more likely to perceive infection with H1N1 influenza as potentially fatal (P < 0.001) (Table 2). Roughly half of the participants (59.5%) rated their personal susceptibility to H1N1 influenza as "low" or "somewhat low," although female students were more likely to perceive their personal susceptibility to H1N1 to be higher (p = 0.002).

### Intervariable Correlations

Having an acquaintance with recent flu-like symptoms was positively correlated with reporting recent flu-like symptoms (P < 0.001), information encountered (P < 0.001), and perceived severity (P = 0.026). Reporting recent flu-like symptoms was negatively correlated with hand washing frequency (P = 0.034) and positively correlated with information encountered (P = 0.038) and perceived personal susceptibility (P < 0.001). Hand washing frequency was positively correlated with perceived effectiveness (P = 0.002) and perceived severity (P = 0.001). Information encountered was positively correlated with perceived severity (P = 0.013). Perceived severity was positively correlated with perceived susceptibility (P = 0.001) (Table 3).

**Table 3:** Correlations with each variable

	Acquaintances with recent flu-like symptoms	Recent flu-like symptoms	Daily hand washing frequency	Information encountered	Perceived effectiveness	Perceived severity	Perceived susceptibility
Acquaintances with recent flu-like symptoms		(+) P < 0.001		(+) P < 0.001		(+) P = 0.026	

Recent flu-like symptoms			(-) P = 0.034	(+) P = 0.038			(+) P < 0.001

Daily hand washing frequency					(+) P = 0.002	(+) P = 0.001	

Information encountered						(-) P = 0.013	

Perceived effectiveness							

Perceived severity							(+) P = 0.001

Perceived susceptibility							

Correlation with each variable was tested using Chi-square for trends

(+): positive correlation, (-): negative correlation

### Factors related to frequency of hand washing

In both univariate and multivariate logistic regression analyses, women were found to wash their hands more frequently than men (Table 4). Participants who perceived hand washing as more effective washed their hands more frequently, as did those who perceived the severity of H1N1 influenza to be higher. However, no significant relationships were identified between hand washing frequency and the following variables: having an acquaintance with recent flu-like symptoms, the degree of information encountered about hand washing, and the perceived susceptibility to infection. Interaction terms were tested, however no interaction effects between independent variables were found. The p-value for the Hosmer-Lemeshow test was 0.465.

**Table 4:** Logistic regression analysis: factors related to recent reported increases in hand washing frequency (≥5 times a day/<4 times a day).

	Univariate		Multivariate*	
		
	OR	95%CI	OR	95%CI
Gender				
Males	1	-	1	-
Females	2.56	1.87-3.52	2.64	1.79-3.90
Acquaintances with recent flu-like symptoms				
No	1	-	1	-
Yes	1.31	1.00-1.72	1.16	0.84-1.60
Information encountered				
No	1	-	1	-
Yes	1.27	0.78-2.09	1.47	0.80-2.70
Perceived effectiveness				
Negligible	1	-	1	-
More or less	1.67	0.77-3.65	2.21	0.72-6.77
Substantial	2.67	1.25-5.71	4.04	1.34-12.15
Perceived severity				
Mild symptoms like common cold	1	-	1	-
Substantial limitation in daily life	1.52	1.13-2.03	1.39	0.99-1.97
Have severe consequences	1.18	0.54-2.54	0.87	0.34-2.25
May die from it	2.32	1.46-3.67	1.77	1.00-3.12
Perceived susceptibility				
Very low	1	-	1	-
Somewhat low	1.18	0.81-1.74	1.10	0.73-1.65
Nor low, nor high	1.00	0.68-1.47	0.79	0.52-1.21
Somewhat high	1.33	0.68-2.57	0.98	0.48-1.99
Very high	0.11	0.31-3.16	1.02	0.29-3.54

* adjusted for gender, acquaintances with recent flu-like symptoms, information encountered, perceived effectiveness, perceived severity, perceived susceptibility

### Relationship between subjects with recent flu-like symptoms and hand washing frequency

In men, flu-like symptoms were more prevalent in participants with less frequent hand washing habits. However, no such relationship between recent flu-like symptoms and hand washing frequency was identified in women. After adjusting for age, residence type, and having an acquaintance with flu-like symptoms by multivariate logistic regression, men with frequent hand washing behavior were found to be less likely to experience flu-like symptoms. However, no such relationship was observed in women. The *p-*value for the Hosmer-Lemeshow test was 1.000 for males and 0.296 for females (Table 5).

**Table 5:** Relationship between recent flu-like symptoms and reported hand washing frequency.

Daily hand washing frequency	Males			Females		
		
	Flu-like symptoms*	**OR**^**†**^	95%CI	Flu-like symptoms	**OR**^**†**^	95%CI
#4	25.6%	1	-	42.5%	1	-
5-7	21.2%	0.78	0.50-1.23	48.0%	1.30	0.68-2.48
#8	12.4%	0.45	0.23-0.90	33.3%	0.64	0.28-1.48

*p-value < 0.05 in chi-square test

^† ^Multiple logistic regression adjusted for age group, type of residence, existence of flu symptoms in participants' acquaintances

## Discussion

Our study demonstrates that, during the peak pandemic periods of H1N1 influenza, most subjects reported increasing their personal frequency of hand washing in order to prevent infection. Similar findings were also reported in studies conducted at the beginning of the H1N1 influenza pandemic in Hong Kong [12]. In a study from the United Kingdom (UK), 28% of subjects reported changing their hand washing behavior as a result of H1N1 influenza [13]. When viewed together, the data from all of these studies imply that behaviors preventative of infection - such as hand washing - become more prevalent during pandemics.

Here, we found that, during the H1N1 pandemic, men reported washing their hands less frequently than women. Several previous studies have also concluded that women are more likely to follow behavioral recommendations (such as hand washing) to prevent the transmission of H1N1 influenza, SARS, or other infectious diseases [7,8,11,13]. Our results also show that the majority of subjects had encountered information about hand washing (males: 91.2%, females: 94.6%) and most perceive hand washing to be an effective measure in preventing the transmission of H1N1 influenza (males: 95.7%, females: 96.1%). These results suggest that the recent public campaigns regarding H1N1 infection prevention conducted through mass media and public education have been successful in terms of public knowledge acquisition. Similar findings were observed in other studies performed during the H1N1 influenza pandemic both in Hong Kong [12] and the UK [13]. However, in the present study, we demonstrate that gender significantly affects perceptions of hand washing effectiveness. Specifically, men are more likely to perceive hand hygiene to be an effective preventive measure against H1N1 infection. Interestingly, several previous studies conducted in Hong Kong during the SARS pandemic concluded exactly the opposite: these studies found that, when compared with women, men were less likely to believe that preventive behaviors were efficacious in controlling SARS [11,14,15]. While such differences could result from differences in study population demographics, profound differences may also exist in perceptions of hand washing between the two countries. Regardless, these conflicting findings highlight the need for additional cross-national comparative studies similar to one previous study conducted during the SARS pandemic [16].

Notably, our data indicate that gender also affects the participants' perceived severity of the H1N1 influenza as well as their own personal susceptibility to the disease. Not only were perceptions of illness severity higher among women, female participants were also more likely to perceive their own personal susceptibility to H1N1 influenza as higher. With regards to these findings, several previous studies conducted during the SARS pandemic [16] also reported similar gender-specific results. As for the perceived severity of illness and perceived susceptibility to H1N1 influenza, this study shows similar perceptions to a study conducted at the beginning of the H1N1 influenza pandemic in Hong Kong [12]; In the Hong Kong study, 64.0% of participants indicated that an H1N1 influenza infection would have "no impact" or "only a minor impact" on their daily life, while 59.7% of men and 42.9% women in our study held these beliefs. Furthermore, only 7.0% of men and 10.3% of women in our cohort, and 10% of participants from the Hong Kong study indicated believing that their personal probability of contracting H1N1 was "high" or "very high."

In this study, study participants who were female, who perceived the overall severity of the illness to be greater and hand washing to be effective in preventing disease washed their hands more frequently. Notably, these data correspond with results from multiple previous studies in regard to the relationship between the frequency of hand washing and female gender [7,8,11,12,17], perceived efficacy of hand washing [12], and perceived severity of the illness [12]. However, information contact was not corrected with hand washing in this study. We contend that due to a ubiquitous media campaign, the public was already well informed about H1N1 influenza (males: 91.2%, females: 94.6%) by the time our survey was conducted, and thus any effect of obtaining information on hand washing behavior was likely minimized. Similar findings were observed in a previous study from the UK [12], where the perceived susceptibility to the illness had no additional effect on hand washing not already documented by previous studies [8-10,12,18].

Lastly, we examined the relationship between hand washing frequency and flu-like symptoms. Although we did not confirm H1N1 infection by viral culture or PCR, we preliminarily identified a relationship between hand washing behaviors and flu-like symptoms in men. However, as this study is cross-sectional in design, we were not able to determine causality between the variables. Nonetheless, despite being unable to confirm a causal relationship between hand hygiene and H1N1 virus transmission, the correlations identified in this study suggest a possible preventive effect of hand hygiene.

This study has notable several limitations. First, we were unable to distinguish H1N1 influenza infection from other upper respiratory tract infections among individuals who reported flu-like symptoms, as we did not use laboratory confirmation techniques. Symptom-based outcomes often lack specificity for influenza virus infections [5,19], as they allow for variable interpretations. In our self-administered questionnaire, 'flu-like symptoms' could be construed differently by different students. However, as the survey period occurred during the peak period of the H1N1 influenza pandemic, public attention and information about H1N1 influenza can be assumed to be high. Consequently, the reliability and validity of using a descriptor like 'flu-like symptoms' may not have significantly skewed our data. Second, even though the relationship between hand hygiene and flu-like symptoms may have mixed effects on H1N1 influenza and the common cold, the contact and inhalation transmission hypotheses are relatively similar for both diseases, suggesting an overall importance of hand hygiene. Furthermore, as the survey was conducted during the peak period of the H1N1 influenza pandemic, the importance of improved hand hygiene should be emphasized. A third limitation is inherent to the study design: the use of convenience sampling - as opposed to random sampling - imposes some inherent selection bias and diminishes the internal validity. To maintain internal validity, we attempted to match the gender ratio of participants in our study with that of the general university population. Our study is further limited by the cross-sectional study design, which prevents the identification of any causal relationships, even though associations between the variables and reverse cause-effect relationships may exist. Lastly, the participants in our study were exclusively composed of students from a single university in Korea. As this does not represent the general Korean population, caution should be exercised when comparing these results with other studies.

Despite these limitations, our study provides invaluable insight into the general perceptions and preventive behaviors relating to H1N1 influenza among young Koreans during the peak of the H1N1 influenza pandemic. Korean students were found to increase their frequency of hand hygiene during the pandemic, and gender differences were apparent in attitudes and behaviors related to the prevention of influenza through the use of hand hygiene. Here, the factors that affected hand washing behavior were similar to those identified at the beginning of the H1N1 or SARS pandemics, suggesting that public education campaigns regarding hand hygiene are effective in altering individual hand hygiene habits during the peak periods of influenza transmission. Further studies conducted during different periods of pandemic influenza transmission are necessary to better describe changing patterns in public perceptions of illness, preventive behaviors, and outcomes of pandemic influenza transmission.

## Conclusion

Korean students increased their frequency of hand hygiene during the pandemic, and gender differences were apparent in attitudes and behaviors related to the prevention of influenza by hand hygiene. Here, the factors that affected hand washing behavior were similar to those identified at the beginning of the H1N1 or SARS pandemics, suggesting that public education campaigns regarding hand hygiene are effective in altering individual hand hygiene habits during the peak periods of influenza transmission.

## Abbreviations

(WHO): World Health Organization; (SARS): Severe acute respiratory syndrome; (UK): United Kingdom.

## Competing interests

The authors declare that they have no competing interests.

## Authors' contributions

JHP contributed to the conception of study and interpretation, and writing the manuscript. HKC participated in the conception of the study and drafting of the manuscript. DYS participated in the design and data collection. SUK participated in the design and statistical analysis. CMH participated in the design, data collection and statistical analysis. All authors read and approved the final manuscript.

## Pre-publication history

The pre-publication history for this paper can be accessed here:

http://www.biomedcentral.com/1471-2334/10/222/prepub

## Supplementary Material

Additional file 1 **Questionnaire**. Click here for file
