# Supplementary material for: Perceptions and behaviors related to hand hygiene for the prevention of H1N1 influenza transmission among Korean university students during the peak pandemic period
Source: BMC Infect Dis. 2010 Jul 28;10:222. doi: 10.1186/1471-2334-10-222 (PMC2922213; doi:10.1186/1471-2334-10-222)
Supplement: Additional file 1 — Questionnaire. [file 1471-2334-10-222-S1.DOC]

Additional file 1: Questionnaire

1. How many times per day did you wash your hands with soap during the previous month?
2. Less than 1
3. 2-4 times
4. 5-7 times
5. 8-9 times
6. More than 10
7. How many times per day did you wash your hands with soap one year ago?
8. Less than 1
9. 2-4 times
10. 5-7 times
11. 8-9 times
12. More than 10
13. Have you seen or heard any information regarding hand washing as a prevention strategy for H1N1 influenza transmission?
14. Yes
15. No
16. Do you consider hand washing to be an effective means of preventing H1N1 influenza infection?
17. Substantial
18. More or less
19. Negligible
20. If you were infected with H1N1 influenza, how great of a burden would that be on your daily life?
21. Mild symptom like common cold
22. Substantial limitation in daily life
23. Have severe consequences
24. May die from it
25. How possible do you believe it is for you to become infected with H1N1 influenza?
26. Very low
27. Somewhat low
28. Nor low, nor high
29. Somewhat high
30. Very high
31. Have you recently experienced any flu-like symptoms?
32. Yes
33. No
34. Do you have any acquaintances who experienced any flu-like symptoms recently?
35. Yes
36. No
37. Your gender
38. Male
39. Female
40. Your birth year:
41. Current type of residence

| 1. University residence halls |
| --- |
| 1. Others |
